# Green toxicology only becomes beautiful through AI

**DOI:** 10.3389/fchem.2026.1801623

**Published:** 2026-05-22

**Authors:** Alexandra Maertens, Thomas Hartung

**Affiliations:** 1 Center for Alternatives to Animal Testing (CAAT), Johns Hopkins Bloomberg School of Public Health and Whiting School of Engineering, Baltimore, MD, United States; 2 Doerenkamp-Zbinden Chair for Evidence-based Toxicology, Johns Hopkins, Baltimore, MD, United States; 3 CAAT-Europe, University of Konstanz, Konstanz, Germany

**Keywords:** AI-driven chemical discovery, decision support, hazard-informed design, mechanistic toxicology, molecular design, read-across, sustainable chemistry, toxicity prediction

## Abstract

Green Toxicology extends the principles of Green Chemistry by embedding toxicological foresight into chemical design, with the aim of preventing hazards before substances reach markets or the environment. Its conceptual pillars—prevention, precaution, life-cycle thinking, and avoidance of regrettable substitutions—align closely with sustainability agendas such as the European Green Deal and the United Nations Sustainable Development Goals. Despite its promise, Green Toxicology has remained largely aspirational, limited by fragmented data, slow regulatory uptake, and reliance on new approach methodologies (NAMs) that still face validation and acceptance hurdles. Artificial intelligence (AI) offers a transformative solution by integrating heterogeneous datasets, enhancing predictive accuracy, and enabling probabilistic risk assessment. Deep learning, natural language processing, and explainable AI can mine legacy studies, link adverse outcome pathways, and design safer chemistries proactively. Coupled with microphysiological systems and omics, AI makes Green Toxicology predictive, human-relevant, and scalable. Together, they form a practical framework for guiding chemical innovation toward sustainability, reconciling industrial productivity with ecological integrity and public health protection.

## 1 Introduction: from green chemistry to green toxicology

The paradigm of Green Chemistry, formally codified by Anastas and Warner in the 1990s, proposed 12 guiding principles to minimize the generation and use of hazardous substances across the life cycle of chemical products (Anastas and Warner, 1998). These principles—ranging from atom economy and the use of renewable feedstocks to safer solvents, energy efficiency, and the design of degradable products—have since spurred innovations in synthetic chemistry, catalysis, and process design (Anastas and Eghbali, 2010; Clark and Tavener, 2007). Despite this progress, the toxicological dimension of chemical design has often remained an afterthought, with hazard assessments typically performed late in product development and relying heavily on protracted animal studies (Maertens et al., 2024).

**FIGURE 1:**
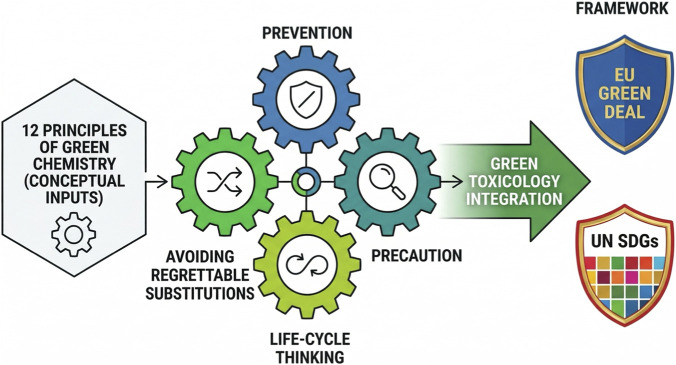
Green Toxicology - the role of toxicology within Green Chemistry to serve sustainability goals Figure manually revised from draft generated with ChatGPT 5.1 and Gemini Nano Banana Pro.

To address this gap, the concept of Green Toxicology (Maertens et al., 2014) was introduced as an extension of green chemistry (Figure 1), embedding toxicological foresight into the earliest stages of molecular design (Maertens et al., 2024; Maertens and Hartung, 2018). Green Toxicology rests on several interlinked principles.

- Prevention, i.e., the proactive design of inherently safer substances that reduce risks before market entry (Crawford et al., 2017; Maertens et al., 2024);
- The precautionary principle, whereby action is justified even in the face of scientific uncertainty about risks (Kriebel et al., 2001);
- Life-cycle thinking, encompassing raw material extraction, production, use, and end-of-life considerations to capture impacts on ecosystems, workers, and consumers (Maertens et al., 2024);
- Avoidance of regrettable substitutions, which have been observed in cases such as bisphenol A analogues or perfluorinated substance replacements, underscoring the need for robust predictive tools (Eladak et al., 2015; Maertens et al., 2021; Tickner et al., 2015).

These pillars collectively reframe toxicology from a reactive to a *preventive* science, integrating 21st century methodologies - including computational toxicology, high-throughput screening, and microphysiological systems - into sustainable chemical design (Maertens et al., 2022; Maertens and Hartung, 2018).

The societal urgency of Green Toxicology is reinforced by major policy frameworks. The European Green Deal explicitly commits to a *“toxic-free environment”* as part of its zero-pollution strategy (European Commission, 2019), while the United Nations Sustainable Development Goals (SDGs) highlight responsible consumption and production (SDG 12) and protection of human health and wellbeing (SDG 3) as central to sustainable development (United Nations, 2015). Green Toxicology thereby becomes not only a scientific strategy but also a governance tool, aligning chemical innovation with international sustainability commitments. In this way, it aspires to reconcile industrial innovation with ecological integrity and public health, catalyzing a shift toward more ethical and environmentally conscious chemical risk management (Hartung, 2023; Kleinstreuer and Hartung, 2024; Maertens et al., 2022).

## 2 The incomplete beauty of green toxicology

While Green Toxicology offers a compelling framework for sustainable chemical innovation, its practical implementation remains constrained by significant scientific and institutional barriers. First, new approach methodologies (NAMs) - including high-throughput *in vitro* assays, *in silico* models, and microphysiological systems (MPS) - still face limited validation (Hartung et al., 2024; Hartung and Kleinstreuer, 2025) and inconsistent regulatory acceptance (Judson et al., 2013; Schmeisser et al., 2023). Without widely recognized standards, uptake remains slow and regulatory submissions still privilege traditional animal-based evidence, even where human relevance may be improved by NAM-based approaches (Hartung and Kleinstreuer, 2025; Schmeisser et al., 2023).

Second, the evidence base available to Green Toxicology is fragmented across diverse data silos, ranging from proprietary industrial dossiers to scattered academic publications and regulatory repositories (Maertens et al., 2014). Although initiatives such as REACH have created one of the largest toxicological data resources (Luechtefeld et al., 2016), much of the information remains difficult to access, harmonize, or reuse in machine-readable form, limiting systematic integration into predictive frameworks (Luechtefeld et al., 2016; Williams et al., 2017). The absence of interoperable data standards hinders the assembly of cohesive knowledge graphs or training sets for computational toxicology. The BioBricks platform is a recent effort to make import functions for more than 90 toxicological databases readily available (Gao et al., 2025).

Third, unresolved trade-offs persist between human health protection, ecological safety, and animal welfare. Disagreements over how much evidence is needed, which endpoints should be prioritized, and how rapidly NAMs should replace animal testing can slow policy harmonization and weaken incentives for rapid adoption of greener approaches (Maertens et al., 2024; Schmeisser et al., 2023).

The *“ugly”* side of Green Toxicology emerges where these gaps intersect. Regrettable substitutions remain a recurring issue, with replacements for bisphenol A or certain per- and polyfluoroalkyl substances demonstrating comparable or newly recognized hazards after widespread adoption (Eladak et al., 2015; Maertens et al., 2021; Tickner et al., 2015). Similarly, NAMs can yield false positives or false negatives when assays are misapplied, poorly standardized, or overinterpreted, leading either to unnecessary alarm or to missed hazards (Kirkland et al., 2007; Schmeisser et al., 2023). Institutional inertia exacerbates these shortcomings, as risk-assessment cultures deeply rooted in animal testing resist disruptive methodologies despite mounting evidence of their limitations (Hartung and Kleinstreuer, 2025; Schmeisser et al., 2023).

**FIGURE 2:**
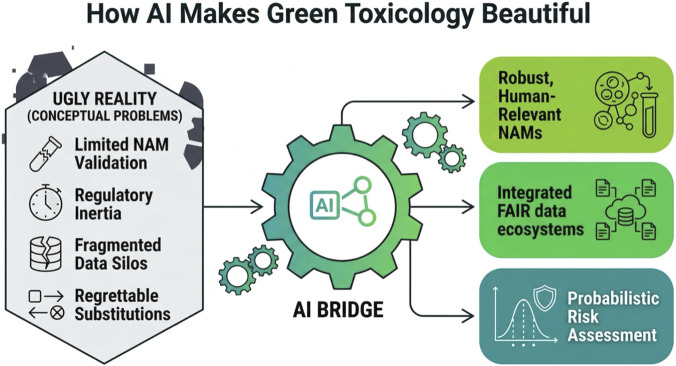
AI as the bridge from the incomplete beauty of Green Toxicology to predictive and sustainable outcomes. The left side depicts persistent implementation barriers - limited NAM validation, regulatory inertia, fragmented data, and regrettable substitutions. The central AI bridge links these constraints to the right side, where integrated FAIR data, human-relevant NAMs, probabilistic risk assessment, safer substitution, and sustainability-aligned decision-making become operational. Figure manually revised from draft generated with ChatGPT 5.1 and Gemini Nano Banana Pro.

In sum, Green Toxicology remains more aspirational than operational (Figure 2) without robust mechanisms to integrate diverse evidence streams into actionable safety decisions. Absent powerful computational tools, its vision risks fragmentation - offering principles without the means for scalable, predictive application (European Commission, 2019; United Nations, 2015).

## 3 The rise of AI in toxicology

The exponential growth of computational power, coupled with the accumulation of large toxicological datasets, has catalyzed a paradigm shift toward artificial intelligence (AI)-enabled approaches in toxicology (Hartung, 2023; Kleinstreuer and Hartung, 2024). Early computational tools in the 1980s and 1990s focused on expert systems such as DEREK, METEOR, HazardExpert, and OncoLogic, which encoded human toxicological expertise into rule-based algorithms for hazard prediction (Benfenati and Gini, 1997). While promising, these systems were limited by rigid rule sets, incomplete knowledge bases, and poor adaptability to novel chemistries.

The 1990s and early 2000s saw increasing adoption of quantitative structure-activity relationship (QSAR) models, regression methods, and support vector machines to link molecular descriptors with toxicity endpoints (Cherkasov et al., 2014). Public repositories such as the OECD QSAR Toolbox and later ToxRefDB and ChEMBL expanded the availability of curated training sets, laying the groundwork for statistical learning. Yet these approaches remained constrained by small datasets, narrow chemical domains, and limited mechanistic interpretability (Cherkasov et al., 2014).

The data-rich era of toxicology emerged in the mid-2000s with the advent of large-scale programs such as ToxCast and Tox21, which together generated tens of thousands of *in vitro* bioactivity profiles across diverse stress-response and receptor pathways (Judson et al., 2014; Kavlock et al., 2008). These datasets transformed toxicology from a largely data-poor discipline into one increasingly suited for AI. High-content imaging, omics technologies, and microphysiological systems (MPS) further expanded the spectrum of measurable endpoints (Marx et al., 2020; Roth and MPS World Summit Berlin, 2019 Consortium, 2021). However, the complexity and heterogeneity of such evidence streams demanded more powerful analytical approaches.

Recent years have seen the maturation of machine learning and deep learning methods for predictive toxicology. Neural networks, including convolutional and recurrent architectures, have been trained on chemical structures, transcriptomic responses, and multi-omics data to predict endpoints such as mutagenicity, hepatotoxicity, carcinogenicity, and developmental toxicity with performance that can rival traditional benchmark models in specific settings (Alam et al., 2025; Challa et al., 2020; Mayr et al., 2016). Deep learning models capture non-linear structure-activity relationships and can provide probabilistic outputs, aligning with the emerging paradigm of probabilistic risk assessment (Maertens et al., 2022). Generative approaches, including adversarial networks and reinforcement learning, are increasingly being explored to design less hazardous chemistries, thereby directly supporting benign-by-design strategies central to Green Toxicology (Alam et al., 2025).

A particularly transformative development is the application of natural language processing (NLP) and large language models (LLMs) to mine legacy toxicological literature, regulatory dossiers, and scientific reports (Corradi et al., 2022; Hartung, 2023). These tools can extract relationships, populate adverse outcome pathways (AOPs), and synthesize evidence at a scale previously impossible. Integration with knowledge graphs further enables the assembly of mechanistic networks linking molecular initiating events to apical outcomes, while explainable AI (xAI) helps identify influential structural features or assay signals, partially addressing concerns about *“black-box”* behavior (Hartung, 2023; Linardatos et al., 2021). Hallucination risk in LLMs has decreased in some constrained workflows, and low single-digit rates have been reported in clinical summarization pipelines, but error rates remain strongly task-dependent and can still be substantial in open-ended settings (Anh-Hoang et al., 2025; Asgari et al., 2025).

By 2025, parts of the broader chemical-safety and materials-modeling ecosystem had also shifted toward graph-based and foundation-style quantitative models that treat chemical or materials structures as dynamic graphs rather than static strings. In toxicology, graph neural networks enriched with toxicological knowledge graphs improved receptor-based toxicity prediction, with the GPS model reaching an AUC of 0.956 on the NR-AR task in Tox21 (Xie et al., 2025). In catalysis and materials science, AQCat25 brought this scale to the foreground, with launch materials describing more than 11 million industrially relevant catalytic interactions and the associated technical paper detailing 13.5 million DFT single-point calculations across 47,000 intermediate-catalyst systems (Allam et al., 2025; SandboxAQ, 2025).

Associated company benchmarks have described speedups of up to 20,000-fold over conventional physics-based catalyst-design workflows; these figures should be interpreted as task-specific engineering benchmarks rather than general toxicology performance metrics. More broadly, the lesson for toxicology is that increasingly large graph-based models can combine speed with high predictive performance, but regulatory usefulness still depends on transparency, reproducibility, and independent validation (Hartung and Kleinstreuer, 2025; Luechtefeld and Hartung, 2025; SandboxAQ, 2025; Tsaioun et al., 2025).

Taken together, AI has moved from the margins of toxicology toward a central enabling technology. It can integrate heterogeneous datasets, improve prediction, reduce reliance on animals, and accelerate evidence synthesis. For Green Toxicology, this integrative capacity is particularly important because it offers a plausible route for translating aspirational principles into operational decision support.

However, significant limitations must be acknowledged. AI models in computational toxicology face challenges related to data quality and availability, as training datasets are often biased toward well-studied chemical classes and endpoints, limiting domain applicability. The *“black-box”* nature of deep learning architectures hampers mechanistic interpretability, a prerequisite for regulatory acceptance. Validation of AI predictions remains difficult, as no consensus framework analogous to OECD test guidelines yet exists for AI-based new approach methodologies. Computational resource requirements are substantial, particularly for large-scale generative models, raising questions of accessibility and environmental sustainability. Furthermore, the gap between promising research-stage performance and formal regulatory acceptance remains wide, with most agencies still requiring traditional evidence for hazard classification (Alam et al., 2025; Hartung and Kleinstreuer, 2025).

## 4 How AI makes green toxicology beautiful

The integration of AI with Green Toxicology helps transform an aspirational concept into an operational paradigm. While Green Toxicology provides the ethical and conceptual framework, AI supplies the analytical tools - scalability, integration, and predictive power - needed to make that framework usable across chemical lifecycles and regulatory contexts (Maertens et al., 2024; Maertens and Hartung, 2018).

First, AI enables predictive design of safer chemicals. Machine learning and deep learning models can identify hazardous structural motifs, quantify structure-activity relationships, and propose novel molecular structures with optimized safety profiles (Alam et al., 2025; Cherkasov et al., 2014; Mayr et al., 2016). Deep generative models are increasingly used to suggest candidate chemistries with reduced toxicity potential, aligning directly with the benign-by-design principle of Green Chemistry (Alam et al., 2025). By guiding synthetic choices before laboratory synthesis, these approaches minimize downstream risk and reduce the likelihood of costly substitutions.

Second, AI facilitates life-cycle integration of exposures and risks, a cornerstone of Green Toxicology. Probabilistic risk-assessment frameworks, supported by Bayesian reasoning and multimodal data fusion, allow hazards to be contextualized within realistic exposure scenarios spanning production, use, and disposal (Kavlock et al., 2008; Maertens et al., 2022; Maertens et al., 2024). This goes beyond traditional hazard identification, capturing trade-offs between occupational safety, consumer use, and environmental fate. Such system-level analyses are essential to avoid burden-shifting, for example, by reducing human toxicity while increasing ecological persistence.

Third, AI amplifies human relevance of hazard testing by bridging *in vitro* and *in silico* data. MPS generate complex phenotypic datasets that can be decoded by machine-learning models and integrated with toxicokinetic reasoning to better emulate human organ responses (Hartung et al., 2025; Marx et al., 2025). High-content imaging combined with AI vision systems enables automated quantitative analysis of cell morphology and stress responses, reducing subjectivity and variability (Tandon et al., 2022). Moreover, NLP tools can mine legacy animal studies, regulatory dossiers, and the broader literature to build mechanistic networks consistent with adverse outcome pathway frameworks (Corradi et al., 2022; Hartung, 2023).

Fourth, AI supports regrettable substitution avoidance, a persistent weakness of Green Toxicology. By systematically screening candidate replacements against large toxicogenomic and *in vitro* datasets, AI can flag analogues with comparable hazard profiles before market introduction (Tickner et al., 2015). For example, machine-learning analyses of bisphenol substitutes and perfluorinated compounds have revealed structural alerts indicative of endocrine disruption and persistence, demonstrating the value of predictive analytics in substitution decisions (Eladak et al., 2015; Maertens et al., 2021; Tickner et al., 2015).

Finally, AI contributes to regulatory translation by enhancing transparency and interpretability. Advances in explainable AI (xAI) allow regulators to understand which structural features or biological endpoints drive predictions, thereby increasing trust in AI-derived evidence (Linardatos et al., 2021). Integration of AI models into initiatives such as the Integrated Chemical Environment (ICE) (Bell et al., 2020), the CompTox Dashboard (Williams et al., 2017), and EU ONTOX (Vinken et al., 2021) enhances accessibility and harmonization, embedding computational methods within existing regulatory workflows. Regulatory traction, however, should not be confused with full regulatory acceptance: OECD test guidelines were developed for experimental methods, and formal criteria for validating AI-based NAMs remain emergent. Concepts such as the OECD (Q)SAR Assessment Framework, ICCVAM’s NAM roadmap, and e-validation illustrate how the field is beginning to bridge that gap, but substantial work remains before AI predictions can routinely substitute for traditional evidence (Hartung et al., 2024; Hartung and Kleinstreuer, 2025; ICCVAM, 2023; OECD, 2023).

In combination, these applications illustrate how AI can make Green Toxicology *“beautiful”* in operational terms (Figure 2): not by aesthetic appeal, but by turning a normative sustainability vision into a practical, predictive, and scalable discipline. Without AI, Green Toxicology risks remaining an elegant but underpowered philosophy; with AI, it becomes a more credible engine for chemical innovation and regulatory modernization.

## 5 Ethical and governance dimensions

While AI promises to elevate Green Toxicology into a predictive and operational discipline, its transformative potential must be tempered by ethical safeguards and governance mechanisms to ensure credibility, transparency, and societal acceptance (Hartung, 2023; Hartung and Kleinstreuer, 2025).

A central challenge is bias in training data. AI models are only as reliable as the datasets they learn from, and toxicological evidence is often heterogeneous, fragmented, and skewed toward legacy animal studies or selective reporting (Hartung, 2023; Luechtefeld et al., 2016). Models trained on biased or incomplete corpora risk perpetuating historical blind spots, reinforcing inequities in regulatory protection, or misclassifying hazards for underrepresented chemical classes. Addressing this requires deliberate curation of datasets according to FAIR principles (Findable, Accessible, Interoperable, Reusable) and incorporation of negative results often absent from the published literature (Wilkinson et al., 2016).

Equally important is interpretability. Deep learning models, despite their predictive power, are often criticized as opaque or difficult to explain (Bender and Cortés-Ciriano, 2021; Linardatos et al., 2021). For toxicology, where mechanistic plausibility underpins regulatory trust, uninterpretable predictions are insufficient. Emerging explainable AI techniques - such as saliency maps, surrogate models, and feature attribution - can help highlight structural motifs, assay endpoints, or exposure scenarios that drive predictions. Such transparency is essential to reconcile AI with established frameworks like adverse outcome pathways and to build confidence among regulators, industry, and the public.

Reproducibility and validation constitute additional governance imperatives. Variability in model development pipelines and opaque algorithms undermine confidence in AI outputs (Bender and Cortés-Ciriano, 2021). Community standards for documentation, benchmarking, and reporting - akin to OECD test guidelines for experimental methods - are needed to anchor AI predictions within regulatory science. Initiatives such as the OECD (Q)SAR Assessment Framework (OECD, 2023) and ICCVAM’s roadmap for new approach methodologies (NAMs) (ICCVAM, 2023) provide promising foundations.

Broader societal concerns also shape the ethics of AI-enabled toxicology. Automated systems may accelerate decision-making but risk eroding accountability if deployed without human oversight (Hartung, 2023; Maertens and Hartung, 2018). Responsible governance requires humans-in-the-loop to evaluate predictions, contextualize results, and prevent overreliance on algorithmic authority. Issues of privacy and data sovereignty further emerge as toxicology integrates exposomic data, biomonitoring, and individual susceptibility profiles. Safeguards for informed consent, anonymization, and equitable benefit-sharing are critical if human-centered data streams are to be responsibly harnessed (Floridi and Cowls, 2019).

Finally, education and cultural change are essential for sustainable adoption. Toxicologists, chemists, and regulators must be trained not only in AI literacy but also in critical appraisal of computational evidence. Multidisciplinary collaboration between data scientists, life scientists, ethicists, and policymakers will be necessary to establish norms that balance innovation with accountability.

In sum, the beauty of AI-enhanced Green Toxicology depends not merely on technical performance but on the trustworthiness of its application. By embedding principles of transparency, fairness, and accountability into governance frameworks, AI can enhance rather than undermine the legitimacy of Green Toxicology in guiding safer chemical innovation (Hartung, 2023).

## 6 A roadmap for AI-enabled green toxicology

The convergence of AI and Green Toxicology provides a unique opportunity to translate sustainability principles into actionable chemical-safety decisions. The roadmap is best understood as a connected sequence in which each layer depends on the previous one and reinforces the next: data ecosystems supply the substrate, AI-enabled NAMs generate interpretable evidence, probabilistic risk assessment turns that evidence into decision-relevant uncertainty estimates, policy alignment governs uptake, and education sustains adoption.

1. Building integrated data ecosystems: The roadmap begins with data, because Green Toxicology can only be as effective as the evidence on which it operates. Large-scale efforts such as REACH, the U.S. EPA CompTox Dashboard, and the Integrated Chemical Environment (ICE) have created unprecedented repositories of toxicological data. Yet much remains fragmented, inconsistently annotated, or locked in proprietary formats. Harmonized FAIR data standards and knowledge graphs are therefore the foundation on which AI-enabled inference must be built (Bell et al., 2020; Gao et al., 2025; Wilkinson et al., 2016; Williams et al., 2017).
2. Embedding AI in NAMs: Once data infrastructures are interoperable, AI becomes the connective tissue across high-throughput screening, omics, and microphysiological systems. Multimodal models can integrate mechanistic and phenotypic evidence, support* in vitro-in vivo* extrapolation, and generate probabilistic rather than binary outputs. This layer converts raw data into actionable biological insight and makes NAM evidence more scalable and human-relevant (Hartung and Kleinstreuer, 2025; Judson et al., 2014; Marx et al., 2025; Tandon et al., 2022).
3. Operationalizing probabilistic risk assessment (ProbRA): The outputs of AI-enabled NAMs should then feed a probabilistic analytical framework. Rather than treating safety as a deterministic yes/no question, ProbRA quantifies uncertainty, variability, and domain applicability, giving regulators and industry a transparent basis for balancing trade-offs across human and ecological endpoints (Maertens et al., 2022).
4. Global and policy alignment: Probabilistic, AI-supported evidence only changes practice when it can be mapped onto regulatory expectations. Examples of AI-supported tools with regulatory traction already exist in ICE, the CompTox Dashboard, and EU ONTOX, but broader uptake still depends on transparent reporting, open science, and validation frameworks that regulators can trust (Bell et al., 2020; Hartung et al., 2024; Tsaioun et al., 2025; Vinken et al., 2021; Williams et al., 2017). OECD guidance on (Q)SAR assessment and ICCVAM’s NAM roadmap are important early building blocks, but formal acceptance criteria for AI-based predictions remain under development (Hartung and Kleinstreuer, 2025; ICCVAM, 2023; OECD, 2023).
5. Cultural and educational transformation: Finally, none of the previous steps will be durable without cultural change. Chemists, toxicologists, regulators, and data scientists need shared literacy in uncertainty, model limitations, and mechanistic interpretation. Education is therefore not an afterthought but the enabler that supports every other element of the roadmap and feeds improvements back into data generation, model development, and policy uptake (Hartung, 2023; Maertens et al., 2024).

**FIGURE 3:**
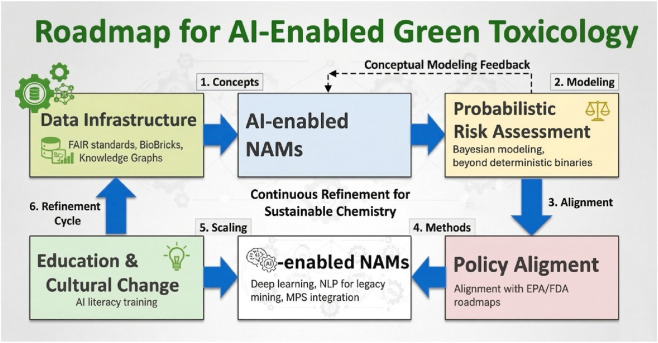
Interconnected roadmap for AI-enabled Green Toxicology. The diagram presents the roadmap as a linked sequence: integrated data ecosystems provide the foundation for AI-enabled NAMs; these in turn support probabilistic risk assessment; outputs then inform policy alignment and regulatory uptake; cultural and educational transformation sustains all stages and feeds back to strengthen the system. Figure manually revised from draft generated with ChatGPT 5.1 and Gemini Nano Banana Pro.

In summary, a roadmap for AI-enabled Green Toxicology must align data infrastructure, methodological integration, probabilistic assessment, policy frameworks, and education as an interconnected system rather than a set of isolated actions. Together, these elements can operationalize the promise of Green Toxicology, ensuring it is not merely a normative aspiration but a practical engine of safer, more sustainable chemistry (Figure 3).

## 7 Conclusion

The vision of Green Toxicology is compelling: to embed toxicological foresight into the design of chemicals and materials so that prevention, precaution, and sustainability become default rather than afterthought. Yet, as outlined, this vision has struggled to move beyond aspiration. Limited validation of NAMs, fragmented data ecosystems, institutional inertia, and unresolved trade-offs between human and ecological priorities have impeded broad adoption.

AI provides the means to overcome these obstacles. By extracting insight from large, heterogeneous datasets, AI enables predictive models that rival traditional animal tests, connect molecular initiating events to apical outcomes, and forecast risks under realistic exposure scenarios. Deep learning and generative methods extend this capability to proactive chemical design, avoiding regrettable substitutions before they occur. Natural language processing (NLP) and knowledge graphs allow the systematic integration of mechanistic and regulatory evidence, while explainable AI (xAI) ensures transparency and interpretability - both essential for regulatory confidence (Hartung et al., 2025).

The *“beauty”* of Green Toxicology thus lies not in its principles alone but in its implementation through AI. Together, they create a discipline that is predictive, mechanistic, probabilistic, and aligned with global sustainability frameworks such as the European Green Deal and the United Nations Sustainable Development Goals (European Commission, 2019; United Nations, 2015). This synergy provides not only a technical solution to chemical risk assessment but also a societal framework that balances innovation with responsibility, human health with ecological integrity, and speed with rigor.

The path forward requires coordinated investment in data infrastructures, validation frameworks, and education, ensuring that chemists, toxicologists, regulators, and data scientists work within a shared ecosystem. With these elements in place, Green Toxicology can evolve from a promising philosophy into a practical engine of safer, sustainable innovation.

In short, Green Toxicology only becomes beautiful through AI: an elegance born not of abstraction but of integration, transparency, and predictive power. Without AI, Green Toxicology risks remaining incomplete; with AI, it holds the promise to transform chemical safety into a driver of sustainability for the 21st century as laid out in the 2025 Stockholm Declaration on Chemistry for the Future (Anastas et al., 2025).
